# Improved Method of Background Value Determination for Sb and Cd in Freshwater Sediment—Insights from Controlling Factors on Spatial Variability

**DOI:** 10.3390/ijerph20054465

**Published:** 2023-03-02

**Authors:** Lingchen Mao, Ruijie Wang, Kai Kang, Feipeng Li, Zheng Zhang, Qingyang Che, Qinyi Tang

**Affiliations:** School of Environment and Architecture, University of Shanghai for Science and Technology, Shanghai 200093, China

**Keywords:** sequential chemical extraction, geoaccumulation index, parent material, sedimentary environment

## Abstract

Variability in the distribution of natural total Sb and Cd in freshwater sediments leads to difficulties in background value (BV) determination. This study aimed to establish a method to determine BV more accurately by investigating the vertical distribution of Sb and Cd in sediment cores collected from a typical river in alluvial plain in China and revealed the factors that control the variation of Sb and Cd BV, which has not been studied in alluvial freshwater sediment. The results suggested that uncontaminated samples for BV calculation should be determined by statistical analysis as human and natural disturbance led to high variation in contamination depth, from <5 cm to >55 cm. The sequential chemical extraction method showed a considerable amount of non-residual fractions of Sb and Cd, which accounted for 48% and 43% of the total, respectively. Acid extractable Cd (16%) was associated to the limestone geology in the area. Fine particles which governed by sedimentary environment contained more natural Sb and Cd, as strong positive correlation was found between clay content and Sb concentration (r = 0.89, *p* < 0.01), as well as Cd concentration (r = 0.54, *p* < 0.01). Based on these findings, a method combined with standard deviation and geochemical method was established to calculate the BV of Sb and Cd, and counter maps were made to cover the variation of BV in the Taipu river sediment. The pollution level has been evaluated by the geoaccumulation index more accurately.

## 1. Introduction

Freshwater sediment is a sink and source of potentially toxic elements (PTEs), including Sb and Cd. It is important for the aquatic ecosystem and human health through a food chain. Researchers have emphasized the importance of an accurate evaluation of contamination level of PTEs in sediment and the ecological risk it brings to the environment [1]. Assessment methods widely used in the evaluation of PTEs contamination in sediments including Potential Ecological Risk Index [2], Geoaccumulation Index [3], Nemerow Synthetical Pollution Index [4] and Enrichment Factor methods [5]. Most of these methods involve the use of the ‘background value (BV)’ of the PTEs in sediment. It is important to determine and use the BV correctly in the environmental evaluation of sediment quality. 

The BV refers to the natural level of element content that is not or is less affected by human activities [6]. It reflects the natural concentration in parent materials and is a key standard to measure the level of pollution [7]. However, existing studies on the BV of PTEs in freshwater sediment mainly focus on main streams and primary tributaries of major rivers, e.g., Yangtze River [8] and Zhujiang River [9,10] in China, Mapocho River in Chile [11] and Tisza River in Serbia [12]. Because of the high variability of BV in sediments [13,14], the absence of regional BV can lead to large bias of pollution assessment results [15]. As BV is the reference in pollution assessment methods, i.e., used as denominator in the calculation, the bias in results is more serious for PTEs with relatively low background value elements such as Sb and Cd. Therefore, the determination of Sb and Cd BV in sediments is essential, especially in areas with intense human activities and multi-contamination sources. 

However, at present, there is no universal or standard method for BV determination in freshwater sediment. Current methods of sediment BV determination can be categorized into three groups including geochemical, statistical and combined methods. Geochemical method is probably the most traditional method to establish BV of PTEs in sediments by removing surface samples exposed to anthropogenic pollution and using the PTE concentrations from “uncontaminated samples”. Samples for this method include shale, deep core, and clay minerals [16,17,18]. The statistical method is based on collecting surface sediment samples after a random or grid layout in the study area. After eliminating outliers, i.e., contaminated samples, in the data set through statistical analysis [19], the mean or median value is set as the BV for the area. The combined method is a combination of geochemical and statistical methods, in which “uncontaminated samples” are collected as samples of background values and statistical calculations are performed to reduce the random variation of the data [20]. These three types of methods have their own advantages and disadvantages. In general, the application of the geochemical method can eliminate exogenous pollution, but is very operationally demanding. It is also difficult to distinguish whether the samples are deep enough to be isolated from human influence [21]. Statistical methods need a large amount of data as support and it is difficult to quantify the contribution of diffuse source pollution [6]. The combined methods can take advantage of the previous two methods and get more scientific and accurate results. However, they demand heavy workloads and high cost. Moreover, the variability of BV in detailed scale, especially in areas with complicated sedimentary environments, increases the requirement of sampling sites [13,14].

Understanding the underlying mechanism that controls the distribution of naturally occurring PTEs in sediments can help to illustrate the BV variation and reduce the number of core samples. Previous studies on PTEs BV in soils and sediments have summarized that the influencing factors include organic matter (OM), particle size, chemical fractionation, and source of PTEs [21]. It was found that the PTE content of uncontaminated sediments was higher in fine particles (clay particles) [21,22]. The concentration of Sb in surface forest soil with high organic matter can reach 9 times that in deep soil [23]. Mao et al. (2019) found that the content of Sb was positively correlated with the content of Fe, Mn, and OM in the sediment column [13]. Correa-Burrows et al. (2021) [11] suggested that the PTEs BV was significantly affected by the parent material in river sediments in a mountainous area. And high PTEs concentration was found precipitates naturally formed as fine particles. However, to our knowledge, there is a very limited amount of research on factors that influence sediment BV in freshwater rivers on alluvial plain. 

Therefore, this study took the sediments of the Taipu River as the research object. This river can be taken as a representative case of an alluvial aquatic water body, with water input from big lakes and many tributaries, typical hydrological conditions and intense anthropogenic influence. The regional BV of Sb and Cd applicable to the Taipu River was established through the comprehensive comparison between different methods, and the influencing factors including particle size, organic matter content, fractionation and sedimentary environment on BV was discussed, so that the scientific basis for accurate assessment of the PTEs pollution level in freshwater sediments can be provided. In addition, the geoaccumulation index method was used to evaluate the pollution level of Sb and Cd in the surface sediments, which provided data support for environmental planning and management in the study area. 

## 2. Materials and Methods

### 2.1. Study Area

**Figure 1:**
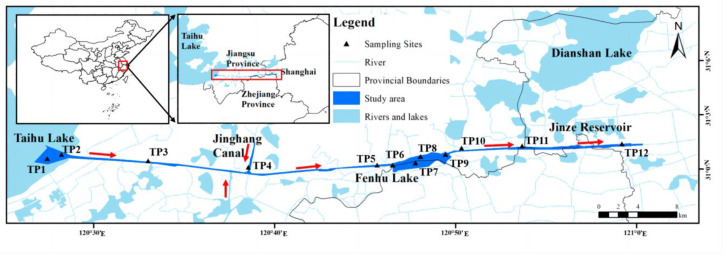
Maps showing the study area. The red arrows demonstrate the major river flow direction.

In this study, we selected the Taipu River as the research area (Figure 1). It is a typical transboundary river in the Taihu Basin in China, connecting two provinces and one city, Jiangsu, Zhejiang and Shanghai. The river is situated in subtropical monsoon climate with average annual precipitation and evaporation around 1091 mm and 1006 mm, respectively [24]. As one of the important artificial rivers in the Yangtze River Estuary, it connects the Taihu Lake and Huangpu River, with water flowing into the river from many tributaries. The Taipu River is 57.2 km long, with an average surface width of 200 m and a water depth of 5.2–8.0 m [25]. The Fenhu Lake, in which the Taipu River flows through, is 6 km long, with a maximum width of 3 km and a depth of 2–5 m. The river intersects with the Jing-Hang canal, which is one of the longest ancient canals in the world. This change in rive topography makes the area an ideal case to study the influence of hydrological conditions and sedimentary environment on the distribution of PTEs [26].

Located in one of the most developed areas in the Yangtze River Estuary, the Taipu River has integrated functions including flood discharge, shipping, landscape and ecology. Lands along the river is densely populated, and there are major pollution sources such as industrial, domestic, and agricultural use, which have a great impact on the water environment quality of the Taipu River [27]. In recent years, many enterprises, mainly textile industry, have gathered on both sides of the river, which increased the pollution load on the regional water environment such as PTEs contamination. From 2014 to 2018, there were 7 accidents of Sb pollution in the Taipu River which led to emergency close of Jinze reservoir intake in downstream [28,29]. Recent studies focused on PTEs contamination in Taipu river sediments. Yao et al. (2022) collected and analyzed surface sediments along the Taipu river and found that Sb and Cd were significantly affected by human activities and were in light-medium pollution status based on the BV in the Taihu Basin, China [30].

### 2.2. Sediment Sampling 

Sampling strategy was made according to river topography, with high sampling frequency at areas with complicated hydrodynamic conditions including inflow, outflow, intersection, and significant increase in width [31,32] (Hånkanson and Jansson, 1983; GB17378.3-2007). Therefore, a total of 12 sampling sites of TP1–TP12 were laid along the Taipu River from East Taihu Lake (upstream) to Jinze Reservoir (downstream), including the upper, middle and lower reaches of the river. As it has been shown in Figure 1, core samples were collected every 7.5 km along the river. Sampling frequency was higher in east Taihu Lake where water flows into the river and in the Fenhu Lake (TP6–TP10). Sampling sites of TP4 and TP11 were allocated at the intersection with main tributaries. 

Sampling of sediment cores was carried out during January to July 2021, using a piston columnar sediment sampler (XDB0204, Beijing New Landmark Soil Equipment Co., Ltd., Beijing, China) The sediment cores were generally grayish black in color and the overall hardness was moderate. The sediment cores were 18–70 cm long depending on the thickness of sediment layer on each sampling site. The cores were further sliced into sub-samples at every 1–3 cm to provide adequate information on Sb and Cd distribution without a waste of operational work. A total number of 309 subsamples were collected. Fragments, stones, residues of plants and animal, and other debris in contact with the sampler were removed. They were stored in polyethylene ziplocked bags and brought back to the laboratory. The collected sediment samples were freeze-dried, gently grounded, and stored in polyethylene bags at −4 °C sieved through 0.15 mm [33] and 0.075 mm [34,35] nylon sieve to analyze organic matter content and total element concentration, respectively. 

### 2.3. Analysis of Total Sb and Cd in Sediments

Before the determination of Sb and Cd in sediments, the freeze-dried samples (<0.075 mm) were pre-treated by mixed acids on hot plate (DigiBlocks36, Lab Tech, USA). 0.2 g of sediment samples were weighed into Teflon digestion tank and a mix of 10 mL HCl, 5 mL HNO_3_, 5 mL HF and 3 mL HClO_4_ was added. Then, it was placed on an electric hot plate at 210 °C for 4 h until the liquid was transparent and clear. The remaining liquid was transferred to a volumetric flask and filtered by a 0.45 μm filter and was analyzed by Inductively Coupled Plasma Mass Spectrometer (ICP-MS, NexION 300X, Perkin-Elmer, Waltham, MA, USA).

### 2.4. Analysis of Sediment Properties 

Sediment particle size distribution was analyzed according to the Code for Marine Survey Part 8: Marine Geological and Geophysical Survey (GB-T 12763.8-2007) [36] by a laser diffraction analyzer (BT9300Z, China), with a measuring range of 0–1250 μm. The sediments was classified according to the Wooden-Wentworth grain size classification scheme which has been widely used (clay: <0.004 mm, silt: 0.004–0.063 mm, sand: 0.063–2.000 mm) [37]. The texture of sediment was classified according to Shepard triangular diagram [38]. The content of organic matter in all samples was estimated on 0.15 mm sieved samples by loss on ignition (LOI in %) at 550 °C for 4 h [33].

### 2.5. Fractionation of Sb and Cd in Sediment

Fractionation information of Sb and Cd were measured on 16 samples in TP7. These samples were selected as 1 from every 3 samples from surface to the bottom of the core. The Wenzel sequential extraction method [39] was used to analyze Sb fractionation, including 5 fractions: non-specifically sorbed, specifically-sorbed, amorphous and poorly-crystalline hydrous oxides of Fe and Al, well-crystalline hydrous oxides of Fe and Al and residual phases. The BCR (European Community Bureau of Reference) sequential extraction method [40] was used to analyze the fractionation of Cd in sediments, including acid-extractable, reducible, oxidizable, and residual fractions. After each stage, the concentration of Sb and Cd in extractants were analyzed by ICP-MS.

### 2.6. Quality Assurance and Quality Control

The reagents used in the experiment were all ultra-pure or at analytical grade. The glassware used in the experiment were all washed following the cleaning procedure of “detergent cleaning—tap water washing—5% nitric acid soaking for 24 h—ultrasonic cleaning—pure water washing—drying”. When measuring Sb and Cd concentrations by ICP-MS, the standard calibration curve of mixed Sb and Cd standard solutions was established (R > 0.9999). The standard reference material for total element concentration analysis in sediment (GBW07455) was purchased from Shanghai Anpu Experimental Technology Co., Ltd. to assess the recovery of the method for total element analysis. The recoveries of Sb and Cd in standard samples were 111.28% and 92.68% respectively, and the RSD of all duplicate samples was less than 12%. The experimental results showed reasonable accuracy and high reproducibility.

### 2.7. Statistical Analysis

The map of study area and contour map showing the distribution of Sb and Cd in sediments were performed using ArcGIS 10.2. The sample data calculation and graphs were completed by Microsoft Excel 2016 and Origin 2019. Pearson correlation and significant difference analysis were conducted by IBM SPSS 22.0. 

## 3. Results and Discussion

### 3.1. Vertical Distribution of Sb and Cd in Sediment Cores

**Figure 2:**
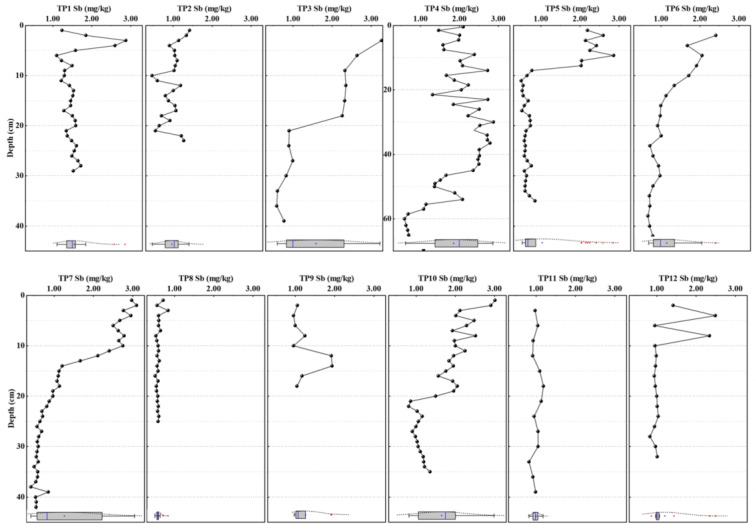
The vertical distribution of Sb in sediment cores sampled in the Taipu River (concentration of Sb (mg/kg): TP1: 1.10—2.87, TP2: 0.46—1.42, TP3: 0.59—3.27, TP4: 0.59—2.88, TP5: 0.50—2.86, TP6: 0.67—2.41, TP7: 0.41—3.10, TP8: 0.51—0.84, TP9: 0.95—1.94, TP10: 0.81—3.01, TP11: 0.82—1.18, TP12: 0.83—2.49).

The vertical distribution of Sb in sediments of the Taipu River is shown in Figure 2. Total Sb concentration in core sediment samples ranged from 0.41 to 3.27 mg/kg. In most cases, the concentration of Sb in surface sediments was significantly higher than that in deep sediments (*p* < 0.05), which indicates the input of anthropogenic Sb to sediments in recent years. With increasing depth, the concentration decreased to a relatively stable level. It can be found that the depth of large fluctuation was different between sampling sites. For example, the concentration of Sb in TP1 and TP2 fluctuated in the depth of 0–5 cm. The highest concentration of Sb in TP1 reached 2.87 mg/kg in the 0–4 cm range, which was about 2.3 times of the lowest concentration (1.24 mg/kg). The fluctuation depth of Sb concentration in TP5, TP6, and TP12 was in the upper 10 cm, while in TP3, TP7, TP9, and TP10 it was about 18–20 cm. The fluctuation depth in TP4 reached to 56 cm. On the contrary, the content of Sb in TP8 and TP11 tended to be stable throughout the whole depth range. It can be explained by the different sedimentary environment along the river [41]. The site of TP4 was sampled at the junction of the Jing-Hang Canal and the Taipu River and TP10 was at the mouth of the Fenhu Lake where there was more significant disturbance in process of sedimentation. The samples at TP8 was collected close to the shore and the hydrodynamic condition was relatively stable [42]. TP11 was sampled in the lower reaches of the Taipu River and the variation trend of Sb content in sediments is similar to that of TP8.

**Figure 3:**
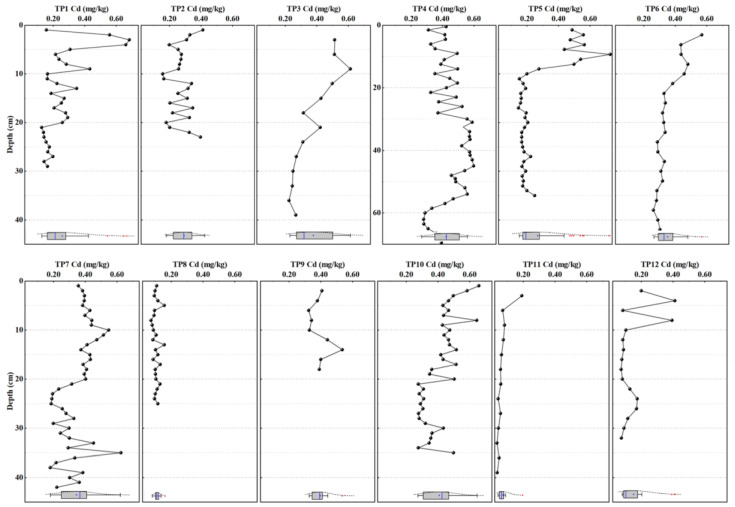
The vertical distribution of Cd in sediment cores sampled in the Taipu River (concentration of Cd (mg/kg): TP1: 0.13–0.68, TP2: 0.16–0.41, TP3: 0.22–0.61, TP4: 0.28–0.60, TP5: 0.15–0.73, TP6: 0.26–0.57, TP7: 0.18–0.62, TP8: 0.07–0.16, TP9: 0.32–0.54, TP10: 0.28–0.66, TP11: 0.03–0.19, TP12: 0.07–0.41).

The vertical distribution of Cd in sediments of the Taipu River is shown in Figure 3. The concentration of Cd in core sediment samples ranged from 0.03–0.73 mg/kg. The variation of Cd content in sediments at most sites of the river was similar to that of Sb, but there were some differences at some points, such as TP7 and TP10. The variation of Cd content in TP7 was relatively stable in the depth of 0–20 cm, while in the deep layer of 20–44 cm was obviously disrupted, and the maximum difference is more than two times. The content of Cd in TP10 also fluctuated in the depth range of 30–35 cm. This implies the historical contamination of Cd in the area. Zhu et al. (2005) [43] found an obvious accumulation of heavy metals including Cd in sediments in the Taihu Basin between 1940 and 1959, which was mainly due to the pollution caused by industrial activities in Wuxi, Jiangsu Province during that period. In contrast, Sb has only been recently added to textile industries in the area in the 1990s [44]. Therefore, Sb concentration was low and stable in deep sediment while high Cd can be found at some locations, i.e., TP2, TP7, and TP10.

Shapiro-Wilk-Test was used to test the normality of the original data at each point, and the results showed that the data distribution types were different among elements. The Cd concentration was normally distributed (*p* > 0.05) at TP2, TP3, TP7 and TP9, while Sb was normally distributed at TP2 and TP11. 

### 3.2. Factors That Affect Natural Distributions of Sb and Cd in Sediments

In order to obtain a more scientific and accurate background value, the chemical fractionation, particle size and LOI in % (mainly organic matter content) in sediments were investigated, so that their influence on Sb and Cd in uncontaminated sediments can be explored.

**Figure 4:**
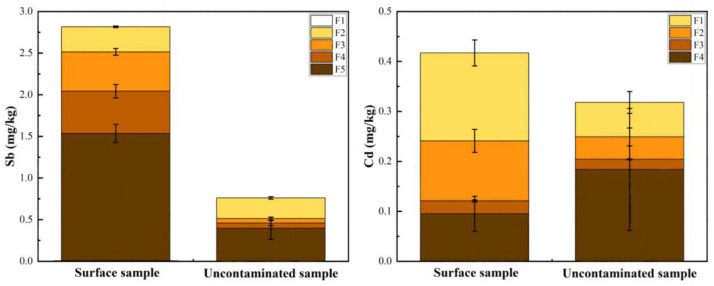
Fractionation of Sb and Cd in 16 samples from TP7 of Taipu River sediments by Wenzel and BCR methods, respectively. The F1, F2, F3, F4 and F5 represent non-specifically sorbed, specifically sorbed, amorphous and poorly-crystalline hydrous oxides of Fe and Al, well-crystalline hydrous oxides of Fe and Al and residual phases in Wenzel, respectively. The F1, F2, F3 and F4 represent acid extractable, reducible, oxidizable and residual fractions in BCR, respectively. Error bars represent the standard deviation of 5 surface layer samples and 11 deep layer samples.

Figure 4 demonstrates the fractionation of Sb and Cd in the sediment core collected at TP7. In general, difference lies not only between surface (0–10 cm) and deep sediments, but also between Cd and Sb because of their geochemical behavior. For Sb, the residual fraction accounted for 54% and 52% of the total in surface and deep sediments, respectively, while the proportion of residual Cd was 23% and 57%. The residual fraction was mainly associated with non-soluble minerals, such as silicate [45]. This fraction is least affected by the environment and is believed to be dominated by natural contribution [46]. Nevertheless, the deep sediments also contain Sb and Cd in other fractions which are more reactive. According to the vertical distribution of Sb in TP7 (Figure 2), deep sediment can be generally regarded as uncontaminated. However, there was also a considerable amount of Sb presented in the specifically-sorbed phase. Dissolution of natural Sb in sediment under reducing conditions may take place [47]. The dissolved Sb can be effectively adsorbed by Fe/Mn (hydr)oxide [48]. Moreover, Oht et al. (2010) [47] also reported natural Sb precipitated with Mn oxides under oxic conditions. This explains the existence but a small proportion of Sb in the crystalline and amorphous oxides fraction in uncontaminated sediments. For Cd, the acid extractable fraction accounted for 16% of the total in deep sediment. This fraction is associated with water-soluble, cation-exchangeable and carbonates [40]. Taihu Lake, which is underlain by limestone, is the main source of the Taipu river water. This leads to Cd primarily presenting in carbonate fraction in sediments [49]. Reducible and oxidizable fraction of Cd were about 29% and 6% of the total. Nevertheless, the non-residual fraction of Cd and Sb is much more abundant in surface sediments than that in deep sediments which proofs that anthropogenic PTEs are usually more reactive. 

In order to reduce the influence of other factors, results of sediment cores collected from the Fenhu Lake was studied (TP6, TP7, TP8, and TP10) to discuss the impact of sedimentary environment to natural Sb and Cd distribution in deep sediments which were uncontaminated or much less contaminated. As it has been discussed above, samples at TP9 were eliminated. Particle size distribution is related to its sedimentary environment. There was a wide range of clay contents in sediment samples down through the core at TP10 (9.7–36.5%), which was located on the river channel. The texture of sediments in TP10 was more scattered in the Shepard triangle diagram (Figure S2). They were mainly clay silt, sandy silt and silty sand. As a channel for flood discharge, the shear velocity of Taipu river water can be high enough to entrain the coarse grain at the flooding period [50]. In contrast, there was less proportion of fine particles in dry season. At other locations, there was a much smaller variation in particle size. The range of clay content in sediment collected from the inlet (TP6) of the lake was 11.2–21.6%. Sediments from this site were silt and clay silt. They were poorly sorted and were likely to be the suspended load [51]. Very low clay contents were found in sediments from the middle of the Fenhu Lake (TP7: 0.63–2.60% and TP8: 2.44–9.77%). There were more coarse particles deposited at these two sites because of the relatively low shear velocity [50]. And there were layers with considerable amount of sand deposited when the flow rate was low. 

**Figure 5:**
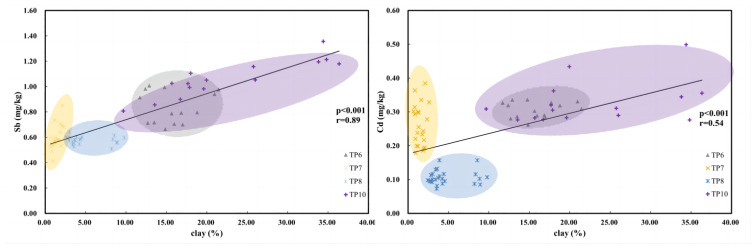
Correlation between PTEs (Sb and Cd) and clay fraction in uncontaminated sediments from TP6, TP7, TP8 and TP10.

Providing the difference in particle size distribution at different locations, its effect on the concentration of natural Sb and Cd were investigated. Figure 5 shows that the clay content was significantly correlated with Sb and Cd in deep samples (*p* < 0.01), with high correlation coefficient of 0.89 and 0.54 for Sb and Cd respectively. This indicates the enrichment of naturally occurring Sb and Cd in fine sediments. High PTEs concentrations were more often found in fine sediments compared with coarse sands, as the former mainly consist of secondary minerals including clay minerals and Fe/Mn (hydr)oxides. These secondary minerals are important binding phases for both Sb and Cd [48,52]. Moreover, Oht et al. (2010) [47] discovered Sb enrichment in fine sediments as a result of natural Sb released under reducing conditions and precipitated with Mn oxides under oxic conditions. This agreed with the fractionation results where there was also a considerable amount of Sb in fractions associated with (hydr)oxides by adsorption and coprecipitation (Figure 4). It should be noticed that the effect of clay content on Cd concentration is weaker than on Sb. This may be explained by the different behavior between the two elements. As Sb is a redox-sensitive element [53], and more easily dissolved and sequestrated by fine components in sediments, while natural Cd is more affected by carbonates as it has been discussed above. Moreover, the results of LOI in sediments suggested a considerate variation in organic matter content (0.57–8.98%, Table S1). The correlation between natural Cd and LOI in %, as an estimation of organic matter content, was significant (*p* < 0.01; r = 0.58, Table S2), which implies the contribution of OM on Cd enrichment.

### 3.3. Comparison of BV Determined by Different Methods

In this study, three kinds of methods were used to calibrate the BV of heavy metals in the sediments of Taipu River, which include geochemical, statistical and combined methods. 

Firstly, in the geochemical method, the BV was established at each sampling point after the removal of surface samples that was subject to anthropogenic pollution and using the average concentration of Sb and Cd in deep uncontaminated samples [54]. Combined with the vertical distribution of Sb and Cd content in the sediment cores (Figure 2 and Figure 3), distinguishing the contaminated and uncontaminated samples is simply based on the criteria of lower Cd and Sb content in deeper sediment than those in the upper layer. It should be noticed that the whole layer of sediment at TP9 was contaminated by Sb and Cd. T-test was used to evaluate the level of difference between deep sediment samples at each sampling site. The results showed that the Sb and Cd concentrations in deep sediments changed significantly along the river (*p* < 0.05; Figures S1 and S2). The BV determined by the geochemical method was presented as 12 threshold values for different locations alongside the river (Tables S3 and S4). 

Secondly, four statistical methods including standard deviation, 2δ Iterative analysis, Grubbs test, and element content boxplot, which have been widely applied in BV determination were also tested in this study. The data used in this method were quoted from Chen et al. [55] (2020), Yao et al. [30] (2022), and the mean value of surface sediments (0—10 cm) in this study. Details of the methods were presented in the Supplementary Materials. When standard deviation and element content boxplot were used, the results were presented as X (median) ± S (standard deviation) [19]. When 2δ Iterative analysis was used, the results were presented as X′(mean) ± S [56]. When the Grubbs test was adopted, the results were different for data of different distribution types. For data conforming to the normal distribution, the results were presented as X′ ± S, otherwise as X ± S [57]. 

Finally, the combined methods were based on the uncontaminated samples. To reduce the influence from random fluctuation of the measured value and potentially contaminated samples in deep sediment layers, for example, Cd in TP2, TP5, TP7 and TP10, the outliers in the data set were further eliminated by statistical methods, i.e., standard deviation, 2δ Iterative analysis, Grubbs test and element content boxplot, as it has been described above and in the Supplementary Materials. Results of distinguishing of contaminated and uncontaminated samples based on standard deviation were shown in Figures S3 and S4 for Sb and Cd, respectively. It should be noticed that, as there were no “uncontaminated samples” in TP9, the BV on that site was not calculated by the geochemical method nor combined method. The results of the combined method were presented as ranged values at each sampling point (Tables S3 and S4). 

In general, the Sb and Cd BVs determined by the geochemical, statistical and combined methods were significantly different. The median of BV of Sb determined by three methods follows the order of statistical (1.55 mg/kg) > geochemical (0.72 mg/kg) > combined methods (0.61 mg/kg). This indicated the diffuse contamination of Sb in the Taipu river surface sediment can generally raise the Sb concentration and lead to a bias of BV determined by statistical methods, even though the outliers were removed [58]. On the other hand, as Sb was only recently added to industries in the surrounding area according to the 2021 yearbook of Wujiang [44], its influence was limited on surface sediments. This made the BV results of Sb generated from the uncontaminated deep samples closer to the true value. Comparison between four statistical calculations shows that there was no significant difference between the results (Figure S5). However, the background threshold obtained by 2δ Iterative analysis is slightly lower than the other three methods. On the one hand, the purpose of iterative analysis is to construct data into a normal distribution and eliminate more outliers in the process of iteration [59]. On the other hand, the average value is used as the final result of this method. Nevertheless, the difference can be covered by the range of BV calculated at each point, i.e., within the range of standard deviation. Therefore, the BV of Sb determined by standard deviation calculated from uncontaminated sediment was chosen under considering the difficulty of data processing, distribution type of data, and the size of standard deviation. 

For Cd, the median of BV determined by three methods follows the order of statistical (0.23 mg/kg) > geochemical (0.11 mg/kg) > combined methods (0.09 mg/kg) in some points (TP5, TP8, TP11 and TP12). The results of comparison among the four types of statistical methods were similar to Sb. Therefore, the methods consistent with Sb was finally chosen to determine the BV of Cd. However, the BV of Cd obtained by statistical methods were generally lower than those obtained by geochemical and combined methods at some sites. Unlike Sb, the usage of Cd has a much longer history in the area in chemical, electroplating, and mechanical industries and agriculture [60]. At some individual points including TP4, TP7, TP9 and TP10, there was no evidence that the Cd concentration decreased to the natural value below a certain depth (Figure 3). Relatively high Cd content in deep sediments at these locations clearly affect the BV determined by geochemical and combined methods (Table S4). 

### 3.4. Assessment of Pollution Level of Surface Sediment Based on Improved BV

The pollution level of Sb and Cd in surface sediments from the Taipu River was evaluated by the geoaccumulation index method (I*_geo_*) [3] as follows:

$$I_{\mathrm{geo}}{=\log}_{2}\frac{C_{i}}{1.5\times \mathrm{BV}}$$

where Ci represents the total concentration of Sb and Cd in surface sediments (mg/kg); BV represents the geochemical background value of Sb and Cd (mg/kg). Three kinds of BV were selected in this study, which included the BV obtained by statistical methods, combined methods, which calculated in Section 3.3, and the BV of soil from Taihu Basin quoted from Liao et al. (2011) [61]. When I*_geo_* was calculated using the BV from the combined method, the value corresponding to each sampling point was selected. The factor of 1.5 was introduced to normalize lithogenic effects [3].

**Figure 6:**
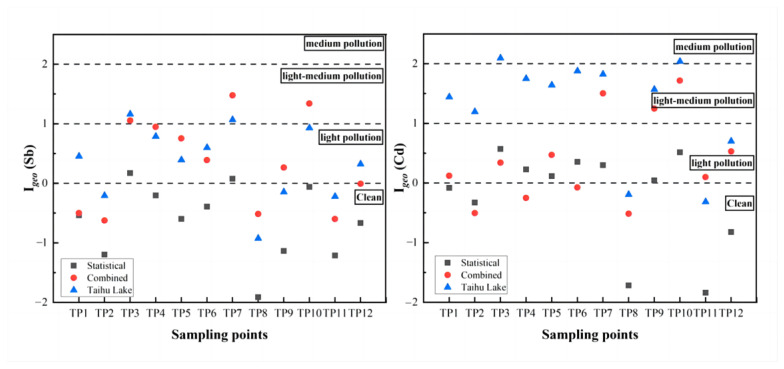
The values of I*_geo_* of Sb and Cd in Taipu River surface sediments, evaluated based on BV calculated from statistical method (standard deviation), combined method (uncontaminated samples + standard deviation) and values quoted from the Taihu Basin [61].

The results were shown in Figure 6. In general, the pollution level was different by using three different BV as basis, except for TP8 and TP11 where there were no Sb and Cd accumulations at the surface (Figure 2 and Figure 3) and I*_geo_* were <0. When the BV obtained by statistical method was taken as the basis, the pollution level of Sb was in the range of clean to light pollution and was clearly underestimated. As it has been mentioned above, this is mainly due to the diffuse contamination in surface sediment in the area. Overestimation of pollution level occurs at TP1 and TP2 using the Taihu Basin BV. Nevertheless, the absolute values of I*_geo_* were significantly different between different BV. For Cd, pollution level was difference between the I*_geo_* calculated from the BV of Taihu plain and that of the combined method. When the BV of Taihu plain was used as the basis, the pollution level of Cd was obviously higher than that of combined methods at TP1–TP6, which was in the range of light-medium pollution. This mainly due to the interference of deep sediment samples in these points when the BV of Cd were calibrated by the combined methods, which leads to the deviation of the results of pollution levels.

In summary, statistical method based on surface sediments providing high BV and led to underestimation of the pollution level. One single threshold value from the Taihu Basin for the whole area can also result in bias in pollution level as it does not consider the variation of the BV.

**Figure 7:**
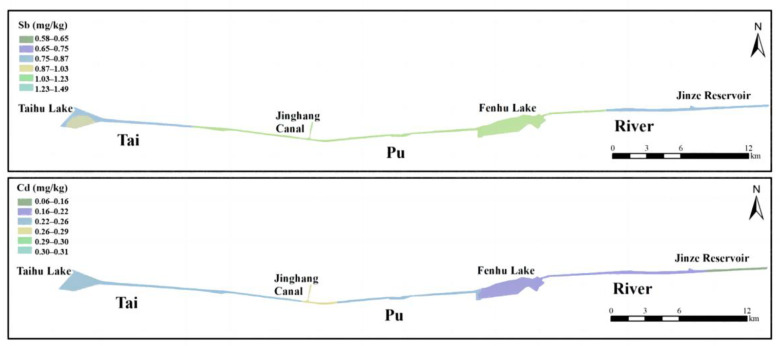
Counter maps of BV of Sb and Cd in the Taipu River sediments.

In the ArcGIS 10.2 geostatistical analysis module, Kriging interpolation was used to make the counter maps of Sb and Cd BV in the Taipu River according to the semi-variance function model (Figure 7). This BV map can successfully cover the variation of naturally occurring Sb and Cd in sediment. It was found that there was high variability in the distribution of BV of Sb and Cd in sediments in the whole study area. The BV of Sb in the east Taihu lake was the highest (1.49 ± 0.13 mg/kg) and showed a trend of gradual decrease along the river. The lowest value (0.58 ± 0.02 mg/kg) was in the middle of Fenhu Lake. The Sb BV in upper stream was likely to be controlled by its natural source. The BV in downstream from the outlet of the Fenhu Lake increased to around 1.04 ± 0.12 mg/kg and was more likely to be dominated by particle size and sedimentary environment. The BV of Cd at the junction of the Jing-hang Canal was the highest (0.32 ± 0.04 mg/kg), which was about 3–5 times of that near Jinze reservoir (0.06 ± 0.01 mg/kg). 

## 4. Conclusions

In this study, based on the spatial distribution of Sb and Cd in sediment cores from the Taipu River region, uncontaminated samples were identified to calculate the background value (BV) of Sb and Cd in sediment. High variability of Sb and Cd was found in deep samples in different locations as a result of combined effect from source contribution, sedimentary environment, particle size distribution and organic matter content. The results of sequential chemical extraction on uncontaminated samples showed that there was considerable proportion of Sb and Cd in non-residual fraction, which accounted for 48% and 43% of the total, respectively. Besides the contribution from the parent material, fine clay and organic matter also led to variation in the BV. In locations with more complicated sedimentary environments, e.g., the inlet and outlet of the Fenhu Lake, there was a large variation in particle size distribution which govern the Sb and Cd in uncontaminated samples. A strong positive correlation was found between clay content and Sb (r = 0.89, *p* < 0.01), as well as Cd (r = 0.54, *p* < 0.01). Based on these findings, a more scientific and accurate method has been established to determine BV, namely the combined method (standard deviation method + geochemical method). Counter maps of BV of Sb and Cd in sediments of the region, drawn by Kriging interpolation, can provide support for the assessment of pollution levels in different river sections. The geoaccumulation index results showed that there was light-medium pollution of Sb and Cd (1 < I*_geo_* < 2) in surface sediments in the upper and middle reaches of the Taipu River.

## Data Availability

The data used to support the findings of this study are available from the corresponding author upon request (mao.lingchen@usst.edu.cn).

## Supplementary Materials

The following supporting information can be downloaded at: https://www.mdpi.com/article/10.3390/ijerph20054465/s1, Figure S1: T-test boxplots of uncontaminated sediment samples Sb and Cd at different points in the Taipu River; Figure S2: Shepard’s sediments type ternary diagram for TP6, TP7, TP8 and TP10 in Taipu River; Figure S3: The vertical distribution of Sb in sediment cores sampled in the Taipu River (mg/kg); Figure S4: The vertical distribution of Cd in sediment cores sampled in the Taipu River (mg/kg); Figure S5: The BV of Sb and Cd determined by statistical and combined methods in Taipu River sediments (mg/kg); Table S1: Summary of organic matter content (LOI in %), clay, silt and sand content (%) in sediment samples collected in the study area (*n* = 309); Table S2: Correlation between PTE contents (Sb and Cd) and clay, silt, sand content (%) and LOI (%) in deep samples from TP6, TP7, TP8 and TP10; Table S3. The BV of Sb determined by different methods in Taipu River sediments (mg/kg); Table S4. The BV of Cd determined by different methods in Taipu River sediments (mg/kg).
