# Supplementary material for: Improved Method of Background Value Determination for Sb and Cd in Freshwater Sediment—Insights from Controlling Factors on Spatial Variability
Source: Int J Environ Res Public Health. 2023 Mar 2;20(5):4465. doi: 10.3390/ijerph20054465 (PMC10001654; doi:10.3390/ijerph20054465)
Supplement: Supplementary file 1 [file ijerph-20-04465-s001.zip › ijerph-2204486-supplementary.pdf]

Supplementary materials

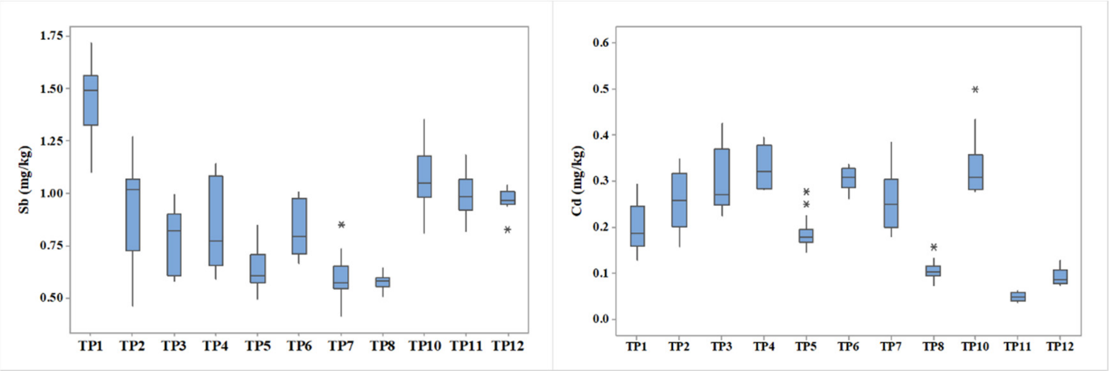

Figure S1. T-test boxplots of uncontaminated sediment samples Sb and Cd at different points in the Taipu River.

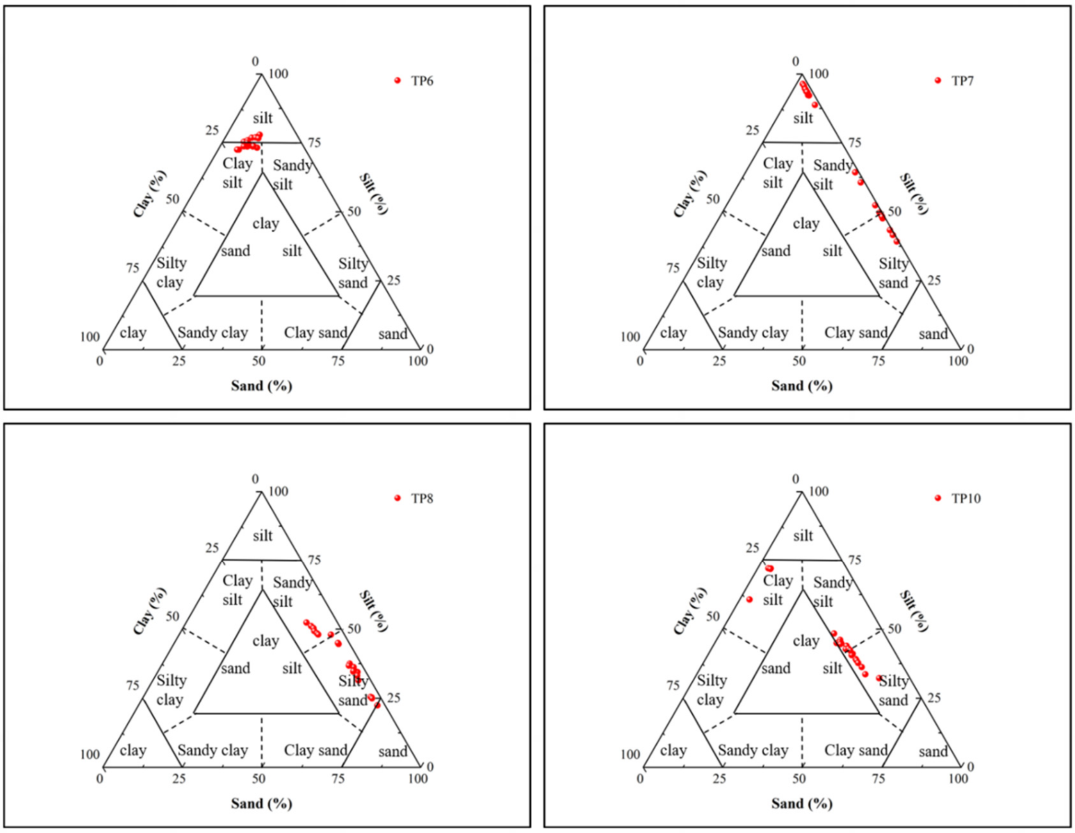

Figure S2. Shepard's sediments type ternary diagram for TP6, TP7, TP8 and TP10 in Taipu River.

Four methods of statistical calculation:

1. Standard deviation

First, the X′ (mean) and S (standard deviation) of the original sample were calculated, and X′ ± 2S was taken as the boundary value to eliminate the data outside the range (outliers). Finally, the X (median) ± S (standard deviation) was taken as the heavy metal background value.

2. 2δ Iterative analysis

The sample data above mean ± 2δ was eliminated by iteration 2δ, and the process was repeated until all the data were between mean ± 2δ. Finally, the X′ (mean) ± S (standard deviation) was taken as the heavy metal background value.

3. Grubbs test

When Grubbs test method is adopted, the original data is first sorted from small to large, where the maximum value is Xmax and the minimum value is Xmin, and then X′ (mean), S (standard deviation) and G values of the data are calculated respectively (G is based on the data volume n and the given significance level, $\alpha = 0.05$ is generally adopted). When Xmax is suspicious, G = (Xmax-X′) / S, when Xmin is suspicious, G = (X′- Xmin) / S. If G > Gα, the maximum or minimum values are considered as outliers and eliminated. And so on until there are no outliers in the data set. Finally, the data distribution type is tested by Shapiro—Wilk test, for data conforming to the normal distribution, the results were presented as X′ (mean) ± S (standard deviation), otherwise as X (median) ± S (standard deviation).

4. Element content boxplot

The data of heavy metal content were used to draw a boxplot, and the data exceeding 95% confidence interval was identified as outliers and removed. Finally, X (median) ± S (standard deviation) was taken as the heavy metal background value.

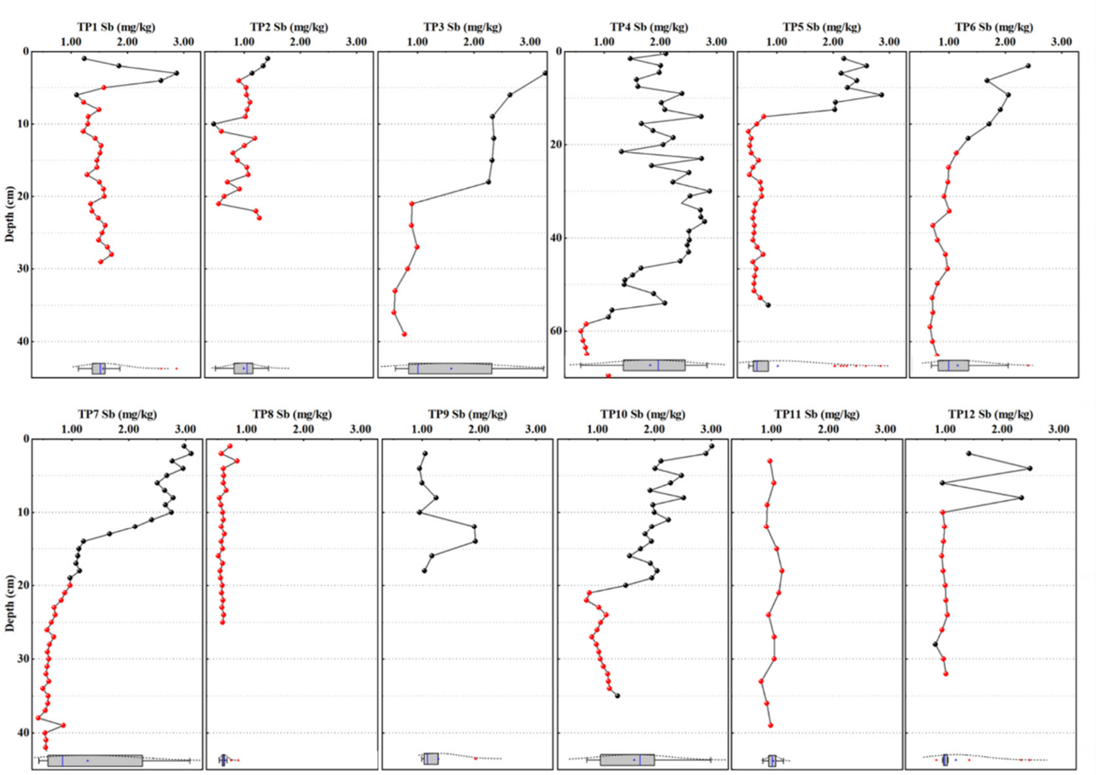

Figure S3. The vertical distribution of Sb in sediment cores sampled in the Taipu River. Black dots represent sediments affected by anthropogenic sources (concentration of Sb (mg/kg): TP1: 1.24—2.87, TP2: 1.14—1.42, TP3: 2.26—3.27, TP4: 1.08—2.88, TP5: 2.03—2.86, TP6: 1.68—2.41, TP7: 1.08—3.10, TP9: 0.95—1.94, TP10: 1.49—3.01, TP12: 0.95—2.49). Red dots represent samples regarded as uncontaminated

sediments (concentration of Sb (mg/kg): TP1: 1.40—1.72, TP2: 0.46—1.27, TP3: 0.59—1.00, TP4: 0.59—1.08, TP5: 0.50—0.85, TP6: 0.67—1.34, TP7: 0.41—0.97, TP8: 0.51—0.84, TP10: 0.81—1.36, TP11: 0.82—1.18, TP12: 0.83—1.04).

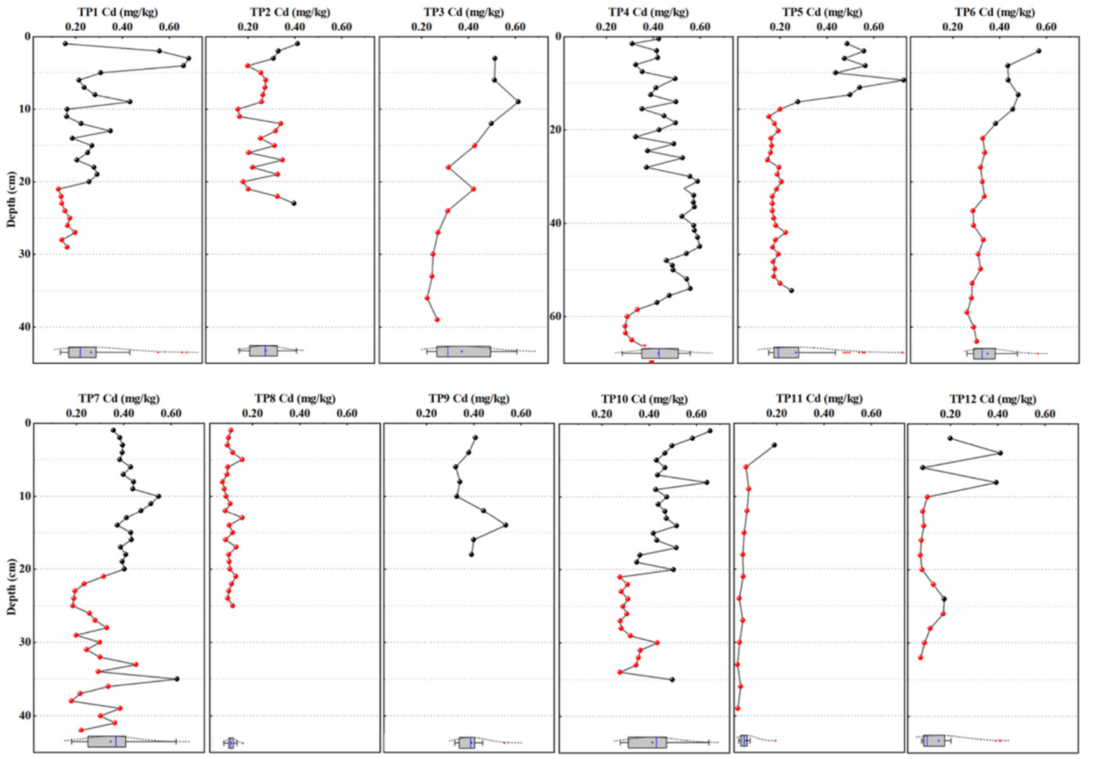

Figure S4. The vertical distribution of Cd in sediment cores sampled in the Taipu River. Black dots represent sediments affected by anthropogenic sources (concentration of Cd (mg/kg): TP1: 0.16—0.68, TP2: 0.31—0.41, TP3: 0.50—0.61, TP4: 0.31—0.60, TP5: 0.44—0.73, TP6: 0.44—0.57, TP7: 0.36—0.62, TP9: 0.32—0.54, TP10: 0.35—0.66, TP11: 0.19, TP12: 0.08—0.41). Red dots represent samples regarded as uncontaminated sediments (concentration of Cd (mg/kg): TP1: 0.13—0.26, TP2: 0.16—0.40, TP3: 0.22—0.43, TP4: 0.28—0.31, TP5: 0.15—0.28, TP6: 0.26—0.38, TP7: 0.18—0.39, TP8: 0.07—0.16, TP10: 0.28—0.50, TP11: 0.03-0.08, TP12: 0.07—0.18).

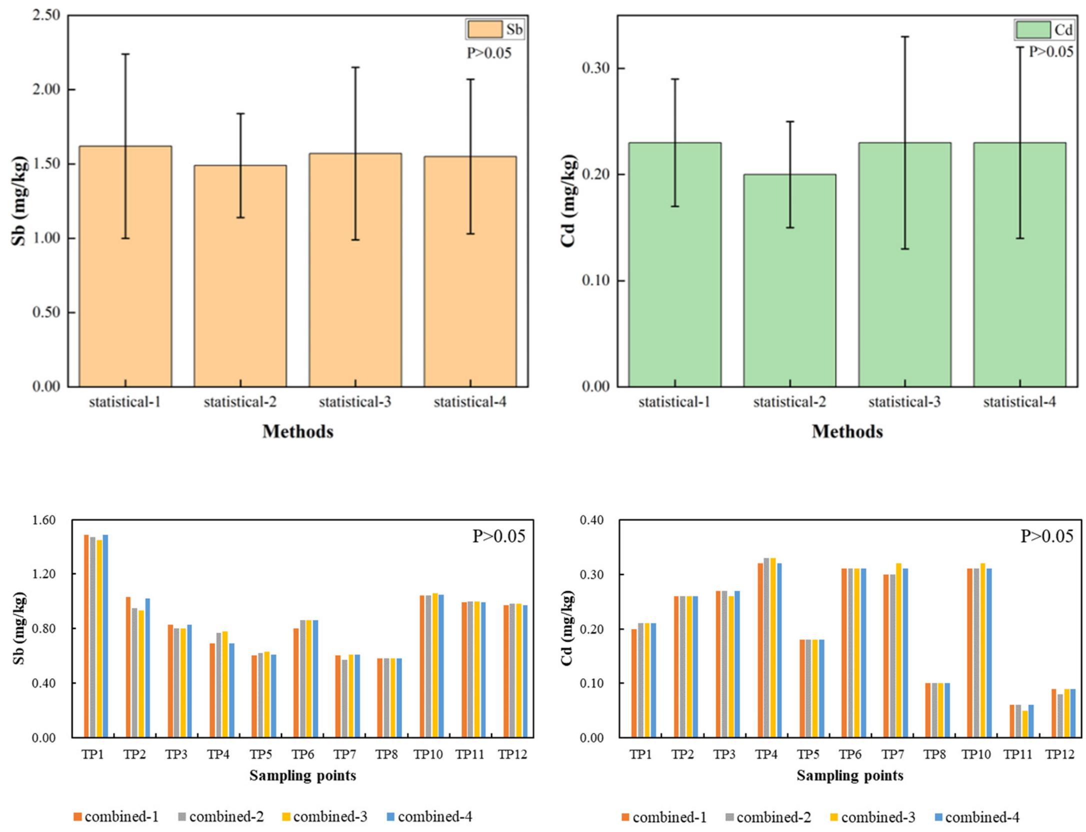

Figure S5. The BV of Sb and Cd determined by statistical and combined methods in Taipu River sediments (mg/kg).

Table S1. Summary of organic matter content (LOI in %), clay, silt and sand content (%) in sediment samples collected in the study area (n = 309).

	**Clay (%)**	**Silt (%)**	**Sand (%)**	**LOI (%)**
Min (%)	0.63	16.04	0.00	0.57
Max (%)	36.35	96.40	80.98	8.98
Mean (%)	15.53	60.74	23.71	3.87
Median (%)	14.88	66.43	8.32	3.79
CV (%)	70.28	30.12	102.29	30.05

Table S2. Correlation between PTE contents (Sb and Cd) and clay, silt, sand content (%) and LOI (%) in deep samples from TP6, TP7, TP8 and TP10.

n = 73	Sb (mg/kg)	Clay (%)	Silt (%)	Sand (%)	LOI (%)
Sb (mg/kg)	1				
Clay (%)	0.89	1			
Silt (%)	0.07	0.01	1		
Sand (%)	0.42	0.41	0.91	1	
LOI (%)	0.38	0.74	0.00	0.30	1
**n = 77**	**Cd (mg/kg)**	**Clay (%)**	**Silt (%)**	**Sand (%)**	**LOI (%)**
Cd (mg/kg)	1				
Clay (%)	0.54	1			
Silt (%)	0.44	0.05	1		
Sand (%)	0.37	0.43	0.92	1	
LOI (%)	0.58	0.74	0.02	0.31	1

Table S3. The BV of Sb determined by different methods in Taipu River sediments (mg/kg).

Sb	TP1	TP2	TP3	TP4	TP5	TP6	TP7	TP8	TP9	TP10	TP11	TP12
Statistical-1						1.53±0.50						
Statistical-2						1.49±0.35						
Statistical-3						1.57±0.58						
Statistical-4						1.55±0.52						
Geochemistry	1.45	0.93	0.80	0.77	0.63	0.89	0.72	0.58	/	1.06	1.00	0.97
Combined-1	1.49±0.13	1.03±0.20	0.83±0.14	0.69±0.16	0.60±0.07	0.80±0.14	0.60±0.21	0.58±0.02	/	1.04±0.12	0.99±0.10	0.97±0.03
Combined-2	1.47±0.13	0.95±0.20	0.80±0.14	0.77±0.16	0.62±0.07	0.86±0.14	0.57±0.02	0.58±0.02	/	1.04±0.12	1.00±0.10	0.98±0.03
Combined-3	1.45±0.15	0.93±0.23	0.80±0.14	0.78±0.16	0.63±0.08	0.86±0.14	0.61±0.22	0.58±0.03	/	1.06±0.14	1.00±0.10	0.98±0.03
Combined-4	1.49±0.15	1.02±0.23	0.83±0.14	0.69±0.16	0.61±0.08	0.86±0.18	0.61±0.22	0.58±0.03	/	1.05±0.14	0.99±0.10	0.97±0.03

1. Standard deviation, 2.2δ Iterative analysis, 3. Grubbs test, 4. Element content boxplot.

Table S4. The BV of Cd determined by different methods in Taipu River sediments (mg/kg).

Cd	TP1	TP2	TP3	TP4	TP5	TP6	TP7	TP8	TP9	TP10	TP11	TP12
Statistical-1						0.23±0.09						
Statistical-2						0.20±0.05						
Statistical-3						0.23±0.10						
Statistical-4						0.23±0.09						
Geochemistry	0.22	0.26	0.29	0.33	0.19	0.31	0.33	0.10	/	0.34	0.07	0.11
Combined-1	0.20±0.06	0.26±0.06	0.27±0.03	0.32±0.04	0.18±0.02	0.31±0.02	0.30±0.09	0.10±0.02	/	0.31±0.04	0.06±0.01	0.09±0.03
Combined-2	0.21±0.05	0.26±0.06	0.27±0.03	0.33±0.04	0.18±0.01	0.31±0.01	0.30±0.02	0.10±0.01	/	0.31±0.03	0.06±0.01	0.08±0.01
Combined-3	0.21±0.06	0.26±0.07	0.26±0.03	0.33±0.04	0.18±0.02	0.31±0.02	0.32±0.09	0.10±0.02	/	0.32±0.07	0.05±0.01	0.09±0.03
Combined-4	0.21±0.07	0.26±0.07	0.27±0.03	0.32±0.04	0.18±0.02	0.31±0.03	0.31±0.11	0.10±0.02	/	0.31±0.04	0.06±0.01	0.09±0.03

1. Standard deviation, 2.2δ Iterative analysis, 3. Grubbs test, 4. Element content boxplot.
